# The role of hepatocyte nuclear factor 4α (HNF4α) in tumorigenesis

**DOI:** 10.3389/fonc.2022.1011230

**Published:** 2022-09-28

**Authors:** Lei Sang, Xingshun Wang, Weiyu Bai, Junling Shen, Yong Zeng, Jianwei Sun

**Affiliations:** ^1^ The Sixth Affiliated Hospital of Kunming Medical University, Yuxi, China; ^2^ Center for Life Sciences, School of Life Sciences, State Key Laboratory for Conservation and Utilization of Bio-Resources in Yunnan, Yunnan University, Kunming, China; ^3^ The Second Affiliated Hospital of Kunming Medical University, Kunming, China

**Keywords:** HNF4α, tumorigenesis, liver cancer, colorectal cancer, transcription factor

## Abstract

Hepatocyte Nuclear Factor 4 Alpha (HNF4α) is a master transcription factor mainly expressed in the liver, kidney, intestine and endocrine pancreas. It regulates multiple target genes involved in embryonic development and metabolism. HNF4α-related diseases include non-alcoholic fatty liver disease (NAFLD), obesity, hypertension, hyperlipidemia, metabolic syndrome and diabetes mellitus. Recently, HNF4α has been emerging as a key player in a variety of cancers. In this review, we summarized the role and mechanism of HNF4α in different types of cancers, especially in liver and colorectal cancer, aiming to provide additional guidance for intervention of these diseases.

## Introduction

HNF4α is a critical transcription factor (TF) during development. Its silencing and dysfunction could lead to stunted development in gastrula formation (1), liver (2) and kidney (3). Interestingly, enforcing expression of HNF4α, in cooperation with Forkhead Box Protein A3 (FOXA3) and Hepatocyte Nuclear Factor 1-Alpha (HNF1α), could even reverse the hepatocellular carcinoma (HCC) cells into normal hepatocyte-like cells (4). Furthermore, HNF4α binds to different gene clusters between undifferentiated state and differentiated state during embryonic development (5), which may be a reason why HNF4α has opposite functions in different types of malignancies.

Another vital role of HNF4α is the regulation of metabolic homeostasis. Most HNF4α-related diseases have abnormal insulin secretion such as occurrence of diabetes mellitus (including Type I and Type II diabetes mellitus), while the underlying molecular mechanism remains elusive (6, 7). It has been shown that HNF4α interacts with Circadian Locomoter Output Cycles Protein Kaput (CLOCK)/BMAL1 to regulate a series of metabolic genes involved in lipid, glucose and amino acid homeostasis. It was known that the circadian rhythm of metabolism was controlled by HNF4α through repression of transcriptional activity of CLOCK/BMAL1 (8). Knockout of BMAL1 attenuated the genome-wide binding of HNF4α in the liver possibly *via* transcriptional downregulation of HNF4α (9). In addition, HNF4α regulated energy metabolism and inflammation through recruiting glucocorticoid receptors (10).

Similarly, HNF4α has been shown to promote glycolysis, glucose uptake, lactic acid production and ATP levels in neuroblastoma cells, and the underlying mechanism involved hexokinase 2 (HK2) and Solute Carrier Family 2 Member 1 (SLC2A1) and the heterogeneous nuclear ribonucleoprotein U (hnRNPU) (11). In pancreatic cancer, HNF4α deletion led to a glycolytic energy metabolism transition from typical pancreatic adenocarcinoma to squamous pancreatic cancer, in which Fructose-Bisphosphate Aldolase A (ALDOA), Hexokinase 1 (HK), and Glycogen Synthase Kinase 3 Beta (GSK3β) genes are upregulated. The downstream Wingless-Type MMTV Integration Site Family, Member 7A (WNT7A) and Protein Kinase AMP-Activated Catalytic Subunit Alpha 1 (AMPK) signal activation further led to drug resistance in squamous pancreatic cancer (12).

Here, we review the role and the mechanism of HNF4α in various cancers, try to emphasize the importance of HNF4α in tumorigenesis and look forward to helping with the treatment and prevention of cancer.

## The expression of HNF4α in different tumors

**Figure 1:**
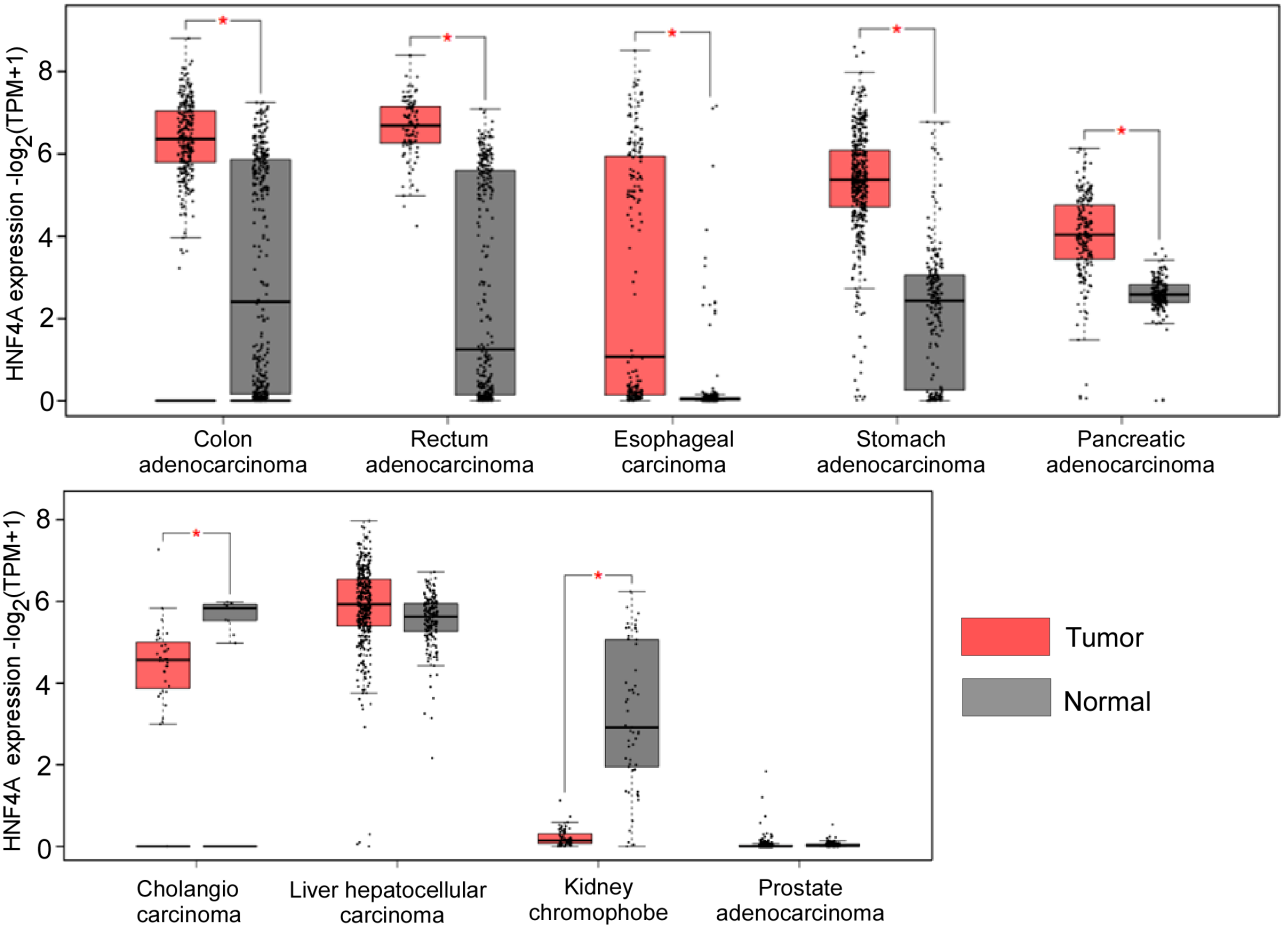
HNF4α expression levels in different types of cancer tissues and their matched normal counterparts. *indicates p<0.05. The raw data are from http://gepia2.cancer-pku.cn.

HNF4α has been demonstrated to be a tumor suppressor in certain types of tumors but act as an oncogene in other forms of cancers. We also analyzed the expression levels of HNF4α in different tumors from the TCGA database and found that HNF4α is upregulated in most gastrointestinal tumor tissues when compared to their matched normal tissues, including colon and rectal adenocarcinoma, esophageal cancer, stomach cancer and pancreatic cancer. However, HNF4α expression level is downregulated in cholangiocarcinoma and kidney chromophobe tissues compared to their normal counterparts (**Figure 1**). Although we did not see significant change of HNF4α expression in HCC and prostate cancer (**Figure 1**), HNF4α plays an important role in HCC and prostate cancer (see below).

## HNF4α function in hepatocellular carcinoma

The important role of HNF4α in development and metabolism, especially in liver tissues, led to the initial research focusing on HNF4α in liver cancer. HNF4α was shown to play an inhibitory role in the development of liver cancer, and was significantly correlated with genes related to drug absorption, distribution, metabolism and excretion in patients with liver cancer (13). Mutations in the Catenin Beta 1 (*CTNNB1*) exon 3 region were detected in 54 of 59 samples (92%) of pediatric hepatoblastoma; In such tumors, Wnt signaling and cell cycle pathways are usually upregulated. Moreover, in more malignant genotypes, HNF4α/CEBPα (CCAAT Enhancer Binding Protein Alpha) binding regions of the genome is highly methylated, and HNF4α/CEBPα transcriptional activity is inhibited (14).

HCC associated with hepatitis B virus (HBV) showed that E2F Transcription Factor 1(E2F1) acts as an active UR (upstream regulator), to positively regulate cell cycle and DNA replication, while HNF4α and HNF1α function as inhibitor URs. In alcoholic HCC, Erb-B2 Receptor Tyrosine Kinase 2 (ERBB2) is activated, while HNF4α and Nuclear Transcriptional Regulator Protein 1(NUPR1 are inhibited) (15).

Taniguchi, H. et al. provided evidence that the HNF4α gene mutations G79C, F83C and M125I (Zn DNA domain) are loss-of-function mutations that would lead to decreased expression of HNF1α and Apolipoprotein B (ApoB) genes and increased risk of liver tumor (16). HNF1α and HNF4α had positive feedback regulation, mutations of Y122C, R229Q and V259F in the POUC domain of HNF1α inhibited its activity and disrupted the binding to the HNF4α promoter, resulting in down-regulation of HNF4α and other HNF1α target genes, as well as disruption of HNF4α-HNF1α transcriptional network, thus triggering the development of HCC (17). In addition, Lysine Demethylase 8(KDM8) is a potential tumor suppressor downregulated in HCC and is a downstream target of HNF4α signaling (18). Furthermore, Mitochondrial Amidoxime Reducing Component 2 (MARC2)-HNF4α forms a positive feedback loop to inhibit the progression of HCC (19).

The expression of hydroxysteroid 17-β dehydrogenase 6 (HSD17B6) in HCC is lower than that in the normal liver and is associated with HCC stage and grade. HNF4α has been shown to bind to the enhancer and promoter regions of the HSD17B6 gene to activate its transcription, and the methylation of the HSD17B6 promoter negatively regulates its expression even in the presence of HNF4α (20).

Accumulating evidence indicates that ferroptosis is closely associated with liver cancer. Zhang, X. et al. showed that HNF4α is a controller of ferroptosis down-regulated factors (FDF), which inhibits iron death by affecting the synthesis of GSH. In response to ferroptosis, dissociation of histone acetyltransferase Lysine Acetyltransferase 2B (KAT2B) blocks the binding of HNF4α to the FDF promoter (21).

For HNF4α-based targeting therapeutics in HCC, HNF4α and HNF1α have been used to inhibit HCC cell proliferation and eliminate tumor-specific features. Takashima, Y et al. showed that the combined transduction of three liver TFs: HNF4α, HNF1α and FOXA3 could stably inhibit HCC cell proliferation and tumor stem cell renewal (22). Oligo-fucoidan, a sulfated polysaccharide, inhibited HCC growth by binding to the Asialoglycoprotein Receptor (ASGR) which led to Signal Transducer And Activator of Transcription 3 (STAT3) phosphorylation, and then p-STAT3 induced the transcription of the HNF4α (23). The mRNA of HNF4α encapsulated by lipid nanoparticles can reduce the liver damage (fibrosis and cirrhosis) in various mouse models. Paraoxonasel is a direct target of HNF4α and participates in the weakening of liver fibrosis mediated by HNF4α (24).

In conclusion, HNF4α maintains homeostasis of liver, and the mutations of HNF4α or epigenetic modifications of promoter regions of its targeting genes lead to the loss of function of HNF4α. Elevated expression of HNF4α has a good prognosis in patients with HCC. In the progression of liver cancer,HNF4α is regulated by factors such as STAT3 and KAT2B, and function as tumor suppressor through HSD17B6, HNF1α, FOXAs, MARC2. Some HCC phenotypes can even be reversed by overexpression of HNF4α.

Interestingly, not all studies support the point that HNF4α inhibits liver cancer. In a recent study on the effect of SNPs in liver cancer, the rs73613962 (T > G) site at the Protein Arginine Methyltransferase 7 (PRMT7) gene has allele-specific enhancer activity. HNF4α preferentially binds to this enhancer region with the risk allele G to activate PRMT7 transcription, and elevated PRMT7 promotes malignant phenotypes of HCC through inhibition of the p53 signaling cascade (25). Another study revealed that HNF4α bound to the -1409 to -1401 region of the circRNA_104075 promoter to induce its expression. Upregulated circRNA_104075 increased the expression of Yes1 Associated Transcriptional Regulator (YAP1), a target of mir-582-3p, by acting as a sponge of mir-582-3p, ultimately promoting the initiation of liver cancer (26).

## HNF4α functions in colorectal cancer

HNF4α also plays a pivotal role in CRC through regulation of several major oncogenic pathways. In 2009, genomic-wide association scanning on 2361 cases of ulcerative colitis and 5417 control cases revealed that HNF4α was related to the progression of ulcerative colitis (27). The Cancer Genome Atlas (TCGA) analysis of colon and rectal tumors showed that HNF4α, TOMM34 (outer mitochondrial membrane translocation enzyme 34) and SRC (non-receptor tyrosine kinase) were overexpressed in colorectal cancer (28).

Expression of P1-/P2-promoter-driven nuclear HNF4α is significantly correlated with cytoplasmic β -catenin in colitis-associated tumor and sporadic CRC. Depletion of HNF4α reduces β -catenin expression (29). HNF4γ, a paralog of HNF4α, and Nuclear Receptor Subfamily 1 Group F Member 3 (RORC) along with HNF4α are also up-regulated in CRC tissues (30). Due to the compensatory role of HNF4γ in intestinal tissues, ablation of HNF4α did not cause changes in HDL level in serum or lipoprotein gene expression in ileum (31). Furthermore, transcription factors such as Heat Shock Transcription Factor 1(HSF1) and Double-Strand-Break Repair Protein Rad21 Homolog (RAD21) play a regulatory role with HNF4α in colorectal cancer metastasis (32). In addition, HNF4α was identified as a positive regulator of oxidoreductase related genes that involved in regulation of ROS level. Knockdown of HNF4α increases and ectopic expression of HNF4α reduces ROS production in CRC cells. Both HNF4α and oxidoreductase related genes are overexpressed in colorectal cancers (33). It has also been found that Nuclear Factor Kappa B Subunit 1 (NF-κB) regulates transcriptional activation of Carboxylesterase 1 (CES1) through HNF4α in invasive CRC (34). These findings suggest that HNF4α functions as an oncogene in CRC.

Contrary to the conclusion above, the expression of HNF4α in colon cancer leads to the decreased expression of oncogenic factors Lysine Demethylase 1 (ALSD1), SET Domain Containing 1A, Histone Lysine Methyltransferase (SETD1A), Protein Arginine Methyltransferase 1 (PRMT1), FOXM1, Protein Tyrosine Kinase 2 (FAK) and Snail Family Transcriptional Repressor 1(SNAI1), and inhibits the tumor-formation of HCT116 cells (35).

To sum up, HNF4α is a key player in CRC while the underlying mechanism is largely unknown. Further investigation of the role and the specific mechanism of HNF4α in the development and progression of CRC is of great significance for establishing HNF4α as a therapeutic target in CRC.

## HNF4α functions in gastric cancer

HNF4α promotes gastrointestinal adenocarcinoma proliferation and survival in a genealogy-specific manner through transcriptional activation of many downstream targets, including HNF1α and interleukin signaling factors (36). The promoter and three distal enhancers of HNF4α are activated by four key transcription factors, ELF3, GATA-Binding Factor 4 (GATA 4), GATA6, and Kruppel Like Factor 5 (KLF5).

HNF4α is highly expressed in both primary gastric cancer and metastasis from gastric cancer to mammary gland, but not in breast cancer, which should be a good marker to distinguish primary and metastatic gastric cancer from breast cancer (37). The same conclusion was confirmed by Saad, DZ. HNF4α was overexpressed in 22 of 23 cases of primary gastric adenocarcinoma and in 15 of 16 cases of metastatic gastric adenocarcinoma, but not in 25 cases of primary breast cancer and 17 cases of metastatic breast cancer, suggesting HNF4α as a valuable biomarker (38). Moreover, HNF4α binds to Mucin 5AC, Oligomeric Mucus/Gel-Forming (MUC5AC) promoter and transcriptionally induces *MUC5AC* expression. Thus, HNF4α correlates with *MUC5AC* mucin expression during stomach development and in GC cells (39).

Furthermore, different HNF4α subtypes derived from two different promoters (P1 and P2) determine the malignancy degree in gastric cancer. Overexpression of P1-HNF4α rather than P2-HNF4α induces tumor growth, and Chemokine Ligand 15 (CCL15) was a direct target of P1-HNF4α in GC (40). In addition, X Inactive Specific Transcript (XIST), a long-strand non-coding RNA, increases enrichment of HNF4α in the promoter region of EPH Receptor A1 (EPHA1), contributing to the deterioration of GC (41).

In conclusion, HNF4α plays a role as a promoter in GC, but underlying mechanism remains elusive, and P1-HNF4α subtype could drive a more malignant phenotype than P2-HNF4α in GC.

## HNF4α functions in other cancers

Apart from HCC, CRC, and GC, HNF4α has been shown to have significant role in many other cancer types.

### Lung cancer

Activation of HNF4α in lung cancer leads to higher lung cancer grade and shorter survival (42). HNF4α has also been found to be elevated in lung adenocarcinoma (43) and induce mucin MUC3 expression in Kirsten Rat Sarcoma Viral Oncogene Homolog (KRAS) mutated lung mucinous adenocarcinoma (44). In addition, HNF4α recognizes the SNP site RS401681, which can interact with Telomerase Reverse Transcriptase (TERT) promoter to increase lung cancer risks (45).

### Pancreatic cancer

Although the TCGA database showed that HNF4α expression level was elevated in pancreatic cancer tissues, Camolotto’s results demonstrated that HNF4α inhibited tumor growth and promoted epithelial development through directly inhibiting expression of Sine Oculis Homeobox Homolog (SIX) 4 and SIX1, two markers of mesodermal/neuronal lineage expressed in basal-like subtypes (46). KRAS(G12D) -driven pancreatic tumors develop after GATA6 deletion, which is accompanied by the loss of HNF1α and HNF4α (47).

### Prostate cancer

It has been revealed that HNF4α-mediated AMPK/mTOR pathway promotes prostate cancer progression (48). A previous study showed that selenium-binding protein 1(SBP1) inhibits prostate cancer growth by reducing oxygen consumption and increasing the activation AMPK, and that HNF4α binds to the promoter of SBP1 to restrain SBP1 transcription (49). In addition, exposure to bisphenol A (BPA) caused prostate preneoplasia, HNF4α-regulated gene networks were altered by BPA, which include nuclear factor-κB, ERK1/2 and insulin-related signaling (50). However, one report indicated HNF4α as a tumor suppressor of prostate cancer by promoting p21-driven senescence (51).

### Renal cell carcinoma

HNF4α function as a tumor suppressor in clear cell renal cell carcinoma (ccRCC). HNF4α has been shown to regulate two metabolic enzymes ABAT and ALDH6A1 leading to inhibition of cell proliferation and migration, and impaired lactate production (52). In addition, HNF4α restrains the development of renal cell carcinoma by transcriptional activation of NR_023387 (53) and inhibition of E- cadherin (54). Moreover, ALDH2 can enhance anthracycline sensitivity of RCC and activates the transcription of HNF4α (55), and HNF4α also increased the chemosensitivity of RCC cells to oxaliplatin and 5-FU (56).

### Cervical, bladder, esophageal and breast cancer

There are few reports about the roles of HNF4α in these tumors. In cervical cancer, HNF4α inhibits the tumorigenic potential *in vivo* and induces the tumor cell G0/G1 arrest through suppression of the Wnt/β-catenin pathway (57). A recent study showed that expression of HNF4α reduced cell proliferation and enhanced cisplatin sensitivity by activation of ALDH6A transcription in bladder cancer (58) and triggered malignant transformation in esophageal carcinoma (59). In addition, upregulation of HNF4α under hypoxia contributes to adriamycin resistance in breast cancer (60).

## Conclusions and future perspectives

The important role of HNF4α in development and metabolism also directly reflect the tumor process. However, the regulation of HNF4α in tumor is not only dependent on metabolic and developmental pathways. In HCC, HNF4α has been demonstrated to inhibit malignant phenotype, while the effect of HNF4α in colon cancer is quite opposite. In addition, a few studies have shown that overexpression of HNF4α promoted the development of gastric and lung cancer and inhibited the development of pancreatic ductal carcinoma. Sporadic studies have shown that HNF4α played a certain regulatory role in all types of tumors. Further investigations of the role of the of HNF4α in different tumor types will greatly enhance the understanding HNF4α biological function and also will be important for development of HNF4α-based therapeutics.

## Author contributions

LS and JWS were responsible for the study concept and design. LS, XW and WB drafted the manuscript. JLS, YZ, and JWS revised and edited the manuscript. All authors contributed to the article and approved the submitted version.

## Funding

This work was supported by National Natural Science Foundation of China (No.82273460 and 32260167), Applied Basic Research Foundation of Yunnan Province (Grant No. 202101AV070002 and 2019FY003030) and Major Science and Technology Projects in Yunnan Province (Grant No. 202102AA310055) and grants (Grant No. 2021Z088 and 2022Y047) from Yunnan University.

## Acknowledgments

We especially thank Dr. Jing Li and Jinquan Cheng for helpful suggestion and the comments on the manuscript.

## Conflict of interest

The authors declare that the research was conducted in the absence of any commercial or financial relationships that could be construed as a potential conflict of interest.

## Publisher’s note

All claims expressed in this article are solely those of the authors and do not necessarily represent those of their affiliated organizations, or those of the publisher, the editors and the reviewers. Any product that may be evaluated in this article, or claim that may be made by its manufacturer, is not guaranteed or endorsed by the publisher.
